# An atypical case of *Babesia bigemina* parasitising a dog from a rural area of eastern Mexico

**DOI:** 10.1590/S1984-29612022039

**Published:** 2022-08-08

**Authors:** José Luis Bravo-Ramos, Sokani Sánchez-Montes, Gerardo Gabriel Ballados-González, Dora Romero-Salas, Jannete Gamboa-Prieto, Angélica Olivares-Muñoz

**Affiliations:** 1 Laboratorio de Parasitología, Posta Zootécnica Torreón del Molino, Facultad de Medicina Veterinaria y Zootecnia, Universidad Veracruzana, Veracruz, México; 2 Facultad de Ciencias Biológicas y Agropecuarias, Universidad Veracruzana, Campus Tuxpan, Tuxpan de Rodríguez Cano, Veracruz, México; 3 Centro de Medicina Tropical, Unidad de Investigación en Medicina Experimental, Facultad de Medicina, Universidad Nacional Autónoma de México, México City, México; 4 Laboratorio de Enfermedades Infecciosas, Facultad de Medicina Veterinaria y Zootecnia, Universidad Veracruzana, Veracruz, México

**Keywords:** Piroplasms, tick-borne disease, phylogenetic analysis, canine babesiosis, Piroplasmas, doença transmitida por carrapatos, análise filogenética, babesiose canina

## Abstract

A dog that shared habitat with domestic animals in a cattle farm and that was exposed to wildlife was taken to a private practitioner for clinical examination. The analyses conducted on the patient revealed the presence of *Babesia bigemina* by a molecular test. Clinical signs such as lethargy, anorexia and hyperthermia > 39 °C, pale mucous membranes and blood urine were observed in the patient. The animal was treated with imidocarb dipropionate (two doses each 0.5 ml/10 kg b.w. at an interval of 14 days). On treatment day 7, the clinical signs were mostly reduced. On day 30, PCR was carried out to assess the efficacy of the treatment, with a negative result. This case represents the first report of babesiosis due to *B. bigemina* in a dog living on a cattle farm in Mexico. It indicates the lower host specify of these pathogens and that dogs can play a role as sentinels of vector-borne parasites in livestock animals.

## Resumo

Um cão que compartilhava hábitat com animais domésticos em uma fazenda de gado e que foi exposto à vida selvagem foi levado a um clínico particular para que fosse examinado. As análises realizadas no paciente revelaram a presença de *Babesia bigemina* por um teste molecular. Sinais clínicos, como letargia, anorexia e hipertermia > 39°C, mucosas pálidas e sangue na urina foram observados no paciente. O animal foi tratado com dipropionato de imidocarb (duas doses cada 0,5 ml/10 kg de peso corporal em um intervalo de 14 dias). No dia de tratamento 7, os sinais clínicos foram reduzidos. No dia 30, foi realizada PCR para avaliar a eficácia do tratamento, com resultado negativo. Esse caso representa o primeiro relato de babesiose por *B. bigemina* em um cão que vive em uma fazenda de gado no México. Isso indica que o hospedeiro inferior especifica esses patógenos, e que os cães podem desempenhar um papel como sentinelas de parasitas transmitidos por vetores em animais de criação.

**Figure 1:**
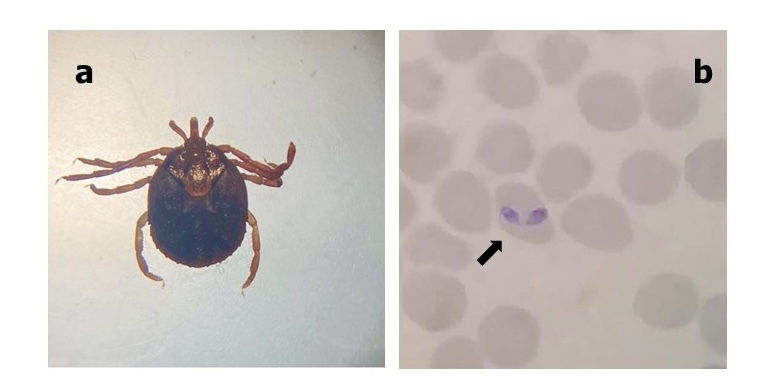
(a) Dorsal view of the female *Amblyomma mixtum*; (b) Photomicrograph of the paired intra-erythrocytic pyriform identified on Giemsa-stained thin blood smear suggested as *Babesia* sp.

**Figure 2:**
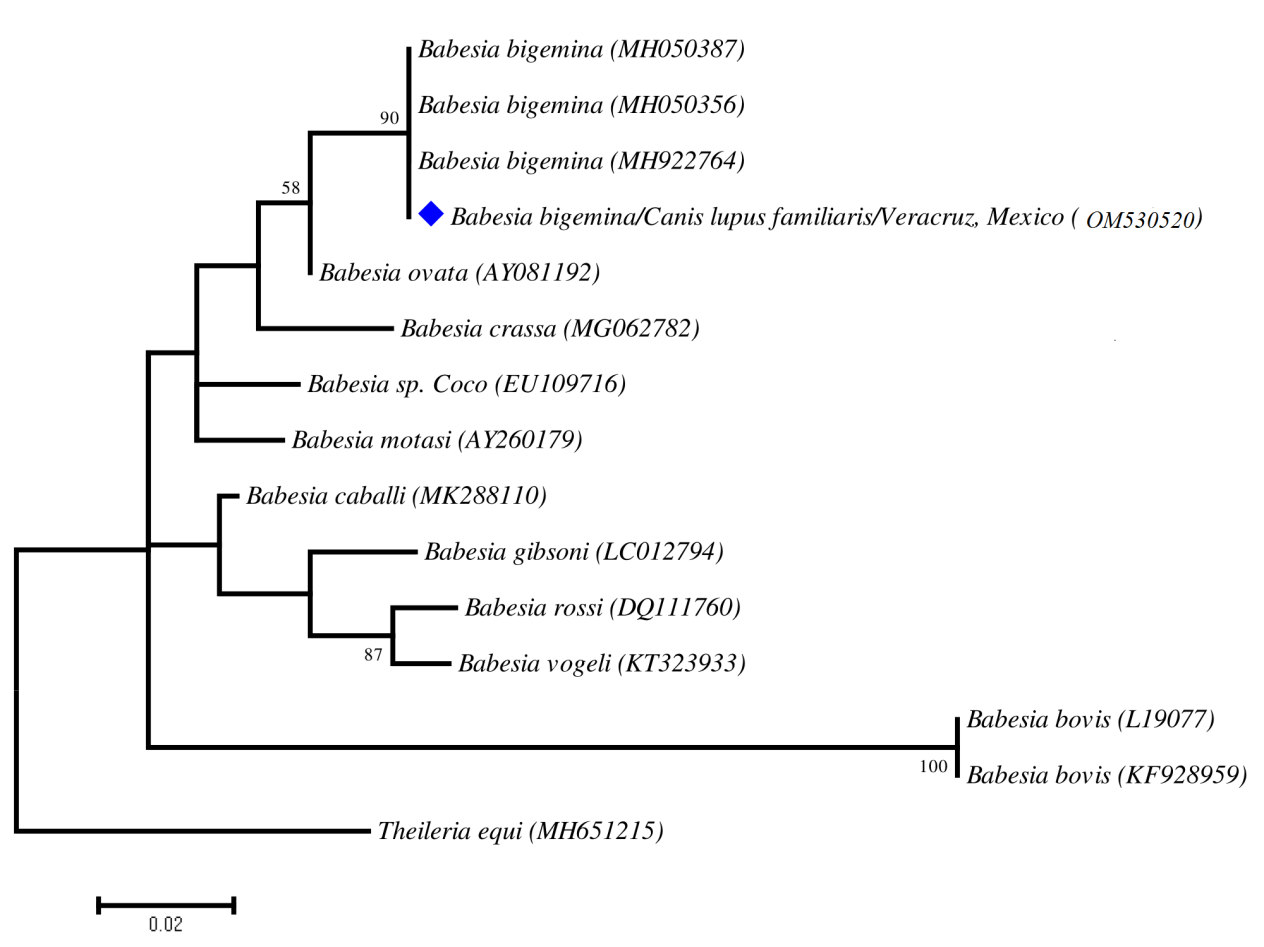
Maximum-likelihood (ML) phylogenetic reconstruction generated with Tamura-Nei model (TN93). Constructed phylogenetic tree based on 571 pb of the *18S rDNA* gene of genus Babesia. Sequence recovered in this study are marked with solid figures (red). The numbers at the nodes correspond to bootstrap values higher than 50% obtained with 1000 replicates. *Theileria equi* was used as outgroup.

Canine babesiosis is one of the most widespread tick-borne diseases which affects dog populations worldwide. The disease may cause a multi-organ dysfunction syndrome, which can compromise the life of the host. It is caused by several species of the protozoan genus *Babesia*. Based on the size of the intraerythrocytic stages of the parasite, two main groups have been recognised: the large group (*Babesia canis*, *Babesia rossi* and *Babesia vogeli*) and the small group (*Babesia gibsoni*, *Babesia conradae*, *Babesia vulpes and Babesia negevi*) (Baneth et al., 2015, 2020; Bawm et al., 2021). Among them, *B. vogeli* is most widely distributed in canids throughout the tropical and subtropical regions (Penzhorn, 2020). It has been postulated that the species of the genus *Babesia* has been considered as highly specific for a given host species or group. However, the specificity of these piroplasms is probably lower than expected (Uilenberg, 2006). Since the implementation of molecular methods, which has increased the sensitivity for the detection of infectious agents, unusual parasite-host associations have started to be described more frequently in dogs. Such is the case of piroplasms from livestock and wild artiodactyls (*Theileria annulata*, *Theileria equi*, *Theileria sinensis*, *Theileria velifera* and uncharacterized *Theileria*), which have been reported in dogs from different countries of the Afrotropical and Oriental regions (Nayyar Ghauri et al., 2019). On the other hand, on cattle farms, canids may share ectoparasites (that act as vectors of several pathogens) with livestock and wildlife. However, the role of dogs as sentinels of tick-borne parasites in livestock is not fully understood. Thus, considering the high prevalence of several tick’s species in canine populations throughout Mexico, it is not surprising to find these shared pathogens among species of hosts that are infested by the same tick species (Ojeda‐Chi et al., 2019). This report is the first record of the livestock piroplasm *Babesia bigemina* infecting a dog. All applicable international, national, and/or institutional guidelines for the care and use of animals were followed. No approval was required. Anesthesia, euthanasia, or any other kind of animal sacrifice was not a part of the study. A 4-year-old male mixed-breed dog that shared habitat with livestock and was exposed to wildlife was taken to a private practice in the municipality of Jesus Carranza (southern Veracruz, Mexico) with a 1-week history of anorexia, pigmented faeces, pigmenturia, fever of 40 °C and lethargy. The dog had not travelled outside of the state of Veracruz and had never received a blood transfusion. After the first physical examination, the dog was evaluated via a baseline assessment consisting on automatic hematological (XT-4000i, Sysmex), and serum biochemistry analyses (Cobas Integra 400 Plus analyzer). Then, a screening for vector-borne diseases, using a immunochromatographic test SNAP**®** 4Dx® Plus followed by a blood smear stained with Giemsa and observed at 100x. Additionally, ticks on the dog´s body were removed using forceps, and each one was kept in a small flask with 70% ethanol. Ticks were identified according to the morphologic characteristics as described elsewhere (Gúzman-Cornejo et al., 2011; Nava et al., 2014). According to taxonomic keys, we identified the tick species *Amblyomma mixtum* on the dog (Figure 1a). Blood and serum analyses revealed anaemia; thrombocytopenia, leucocytosis, eosinophilia, blood urea nitrogen and creatinine levels were increased (Table 1). The SNAP 4Dx test was negative. However, light microscopy of the blood smear revealed the presence of pyriform structures inside erythrocytes (Figure 1b). To confirm the identification of piroplasms, a fragment of the 571-bp region of the 18S ribosomal gene (*18S-rDNA*) was amplified with the primers BAB01 (5′CCGTGCTAATTGTAGGGCTAATACA3′) and BAB02 (5′GCTTGAAACACTCTARTTTTCTCAAAG 3′) as described previously (Almeida et al., 2012; Sarma et al., 2019). Genomic DNA was extracted from 100 μL of whole blood using the QIAampDNA Minikit (Qiagen, Germany). Amplification product were submitted for purification and sequencing at Macrogen Inc., Korea, and the obtained sequence was deposited in GenBank under accession number: OM530520. Subsequently, the sequence was compared with those available in GenBank using the Basic Local Alignment Search Tool (BLASTn tool). The BLAST analysis revealed the highest nucleotide identity (i.e.,100% and 99.2%) with reference sequences of *Babesia bigemina* deposited in GenBank (OK605299, MH050387) reported in cattle and tick *Rhipicephalus microplus* from China and India. Additionally, sequence generated in this study and those of other *Babesia* species deposited in GenBank were aligned using the algorithm MUSCLE in MEGA 11. Subsequently, a phylogenetic reconstruction using the Maximum likelihood method was performed, also in MEGA 11 (Kumar et al., 2018). Branch support was estimated using 1,000 non-parametric bootstraps. The phylogenetic analysis supported the finding of *B. bigemina*. The sequence generated in this work were grouped with those of *B. bigemina* in a monophyletic group with support values that varied from 95-100 (Figure 2).

**Table 1:** Hematological and biochemical analyses in a dog with *B. bigemina* infection during treatment with imidocard dipropionate.

	Parameters	Reference value	Day 1	Day 20
**Hematology**	RBC × 10^6^ /μL	4.5-7.1	3.2	6.5
	White Blood Cells	7.0-10.0	12.2	8.9
	Hb g/dl	11.3-15.5	9.2	13.2
	Ht (%)	50.2-55.6	48.2	52.2
	PLT ×103 /μL	148-450	132.5	146.6
	Eosinophils %	0-2	12	1
	Eosinophils ×10^3^ /μL	0-0.8	2.9	0.4
**Biochemical**	Blood Urea Nitrogen (mg/dl)	**25-30**	32	30
	Creatinine (mg/dl)	0.6-1.2	1.5	1.3
	Total bilirubin (μmol/l)	0.0-0.3	1.1	0.3

The dog was treated with imidocarb dipropionate (two doses each 0.5 ml/10 kg b.w. at an interval of 14 days, intramuscularly) and fluid therapy. Additionally, a single dose of a chewable tablet that contained afoxolaner (2.5-5 mg/kg) and milbemycin oxime (0.5-1 mg/kg) NexGard Spectra ® was given orally. On day 7 of treatment, clinical signs in this patient were mostly reduced, with a rapid resolution. On day 20, the dog underwent physical examination. Red blood analysis showed increased values in RBC, Hb and HT compared to day 0, indicating recovery from anaemia. In day 30, a PCR was carried out again to assess the efficacy of the treatment, and *Babesia* DNA was no longer detectable. The treatment showed good tolerance and safety, with scarce adverse events observed. The case resolved favorably, and the patient was discharged.

In this report, we describe the first record and characterisation of *B. bigemina* in a dog with clinical and haematological abnormalities suggestive of Babesia infection in Mexico. Previous records in Mexico have shown that dogs in rural areas can be infested by several hard tick species such as *Amblyomma mixtum*, *Amblyomma ovale*, *Amblyomma parvum*, *Ixodes affinis*, *Rhipicephalus microplus*, *Rhipicephalus sanguineus* s.l. and *Amblyomma oblongoguttatum*. The tick *A. mixtum*, detected in this case, is the same as that previously reported attached to dogs in rural communities in Yucatan (Ojeda‐Chi et al., 2019). Additionally, *A. mixtum* parasitises a wide range of hosts and can take advantage of the nutritional resources present in the area (Gúzman-Cornejo et al., 2011). For this reason, it is likely that this tick can be a potential transmitter of *Babesia* species and exchanges several pathogens between domestic animals and wildlife.

One of the main clinical findings observed in canine babesiosis is anaemia. Some reports state that oxidative stress and lipid peroxidation play a role in the pathogenesis of anaemia in some hemoprotozoan diseases (Maharana et al., 2016). In biological membranes, these mechanisms induce disturbances of the structural integrity, leading to the rupture of the membrane and the release of red blood cell contents, which causes lysis (Holovakha et al., 2018). However, in babesiosis anaemia is caused by multifactorial components, including direct parasite damage to the erythrocyte membrane, splenic removal of damaged and parasitized erythrocytes, as well as activation of the immune system such as complement cascade and/or presence of anti-erythrocyte antibodies (Köster et al., 2015). Other clinical signs of canine babesiosis include fever, anorexia, depression and lethargy (Crnogaj et al., 2010), which coincide with our case. The abnormalities in the elevation of white blood cells could be related to the *Babesia* infection, with the likely exception of the peripheral eosinophilia. However, absolute eosinophilia cannot be considered a marker of babesiosis, considering that it is not a specific alteration of the infection in dogs but a common finding secondary to several helminths parasitic or allergic diseases (Chidumayo, 2018). Low platelet count is well described in canine babesiosis as well as in other hemoprotozoan diseases, such as malaria and trypanosomiasis (Karasová et al., 2022). However, the mechanism of thrombocytopenia seen in babesiosis is not clear. The severity and rapid recovery of the platelet counts has led to the suggestion that immune-mediated mechanisms are involved (Rautenbach et al., 2018). Higher serum urea levels are not uncommon in dogs with babesiosis, and this elevation is often disproportionate to the rise in creatinine levels. This is probably due to increased protein catabolism and urea production resulting from gastrointestinal haemorrhage or protein catabolism due to the febrile inflammatory illness (Ullal et al., 2018). Regarding treatment, imidocarb is the one of the main drugs used in the antiprozoal treatment of babesiosis and has direct action against the parasite DNA, causing nucleic acid damage and inhibition of cellular repair and replication; however, a few drugs and drug combinations are used in the treatment of canine babesiosis, often without complete parasite elimination, and the dogs usually become chronic carriers or present with recurrent episodes of acute infection (Baneth, 2018). In this new scenario, veterinarians should be aware of unexpected *Babesia* species in dogs that, as known for this species, may be even worse than in livestock, considering the rapid manifestation of the clinical signs. In conclusion, the spectrum of *Babesia* pathogens that infect dogs is gradually being elucidated with the aid of new molecular techniques and meticulous clinical investigation. Additionally, chemoprophylaxis should also be adopted for dogs living in an endemic area, with the primary means of prevention being tick control.
